# Postoperative changes of wrist-brachial index following arteriovenous fistula implantation correlate with steal syndrome, a prospective study

**DOI:** 10.1016/j.jvscit.2025.102002

**Published:** 2025-10-10

**Authors:** Navid Hajihoseini, Mohammadali Vakili, Alireza Aghili, Nazanin Musapour, Pezhman Kharazm

**Affiliations:** a Clinical Research Development Center, 5th Azar Medical Center, Golestan University of Medical Sciences, Gorgan, Iran; b Health Management and Social Development Research Center, Golestan University of Medical Sciences, Gorgan, Iran

**Keywords:** Hemodialysis, Wrist-brachial index, Steal syndrome, Arteriovenous fistula

## Abstract

Arteriovenous fistula (AVF) is the preferred vascular access for hemodialysis in end-stage renal disease. Steal syndrome, a complication causing limb ischemia, may follow AVF implantation. This study evaluated whether postoperative wrist-brachial index (WBI) changes predict steal syndrome, including severe cases requiring intervention. In a prospective study, patients undergoing first-time AVF implantation in 2022 were enrolled. WBI was measured preoperatively, immediately postoperatively, at 2 weeks, and at 3 months. Steal syndrome symptoms (pain, pallor, coldness, numbness, and paresthesia) were assessed at follow-ups. Data were analyzed using the Shapiro-Wilk, *t* test, and Mann-Whitney *U* tests (*P* < .05). Of 59 patients (31 men, 28 women; mean age, 56.9 ± 12.6 years), 23 (39%) had temporary steal syndrome, 16 (27.1%) had prolonged steal syndrome, and 3 (5.1%) required vascular reconstruction for severe ischemia. Immediate postoperative WBI changes (mean of 64.05% in severe cases vs 22.10% overall) were significantly associated with steal syndrome (*P* = .02), including severe cases (*P* = .03). Associations at 2 weeks (*P* = .14) and 3 months (*P* = .11) were not significant. WBI changes correlated with hypertension and smoking but not age, sex, dialysis duration, diabetes, or body mass index. Immediate postoperative WBI changes reliably predict steal syndrome, including severe cases, after AVF implantation. Close monitoring is essential for patients with significant WBI reductions.

End-stage renal disease is defined as irreversible failure of kidneys function, which leads to death without renal replacement therapy.^1^ Renal replacement therapy includes renal transplant (as the most effective and best quality of life), hemodialysis, and peritoneal dialysis.^2^ Most end-stage renal disease patients are managed with hemodialysis. An effective hemodialysis is dependent on functional vascular access to provide sufficient flow to the hemodialysis machine.^3^ An arteriovenous fistula (AVF) is the preferred vascular access according to clinical guidelines.^4^ AVF creation involves anastomosing a superficial upper limb vein (eg, cephalic, basilic) to an adjacent artery, promoting vein dilation and arterialization for repeated punctures.^5^, ^6^, ^7^ Steal syndrome, a complication of AVF, results from arterial flow diversion to the venous system, causing limb ischemia.^8^ Although mild steal syndrome is common, severe ischemia threatening tissue loss occurs in 1% to 10% of patients.^4^^,^^9^ Severe cases may present with pain, pallor, pulselessness, or absent Doppler signals, requiring AVF closure.^10^^,^^11^ The determination of a single reliable test to predict the steal syndrome after AVF implantation has been the subject of several studies.^3^^,^^12^, ^13^, ^14^ The wrist-brachial index (WBI), defined as the ratio of wrist to brachial artery pressure, is a noninvasive predictor of arterial insufficiency.^15^^,^^16^ The WBI is normally about 1, and a preoperative WBI of <0.6 contraindicates AVF creation.^17^^,^^18^ Yet, steal syndrome, including severe cases, can occur in limbs with a normal WBI.^19^ Postoperative WBI changes may better predict ischemia.^12^^,^^20^ This study investigated the correlation between postoperative WBI changes and steal syndrome, including severe ischemia requiring intervention, in AVF patients.

## Methods

This prospective study enrolled 94 patients scheduled for first-time AVF implantation using the brachial artery and superficial veins (cephalic, basilic, or median cubital) in 2022. Only patients with first-time AVF implantation using the brachial artery were included. All patients were excluded from distal AVF implantation because of inappropriate distal vessels. According to the guidelines, our preference is always to place distal fistulas when feasible. Exclusion criteria included unsuitable vessels, left ventricular ejection fraction of <30%, inadequate superficial veins (requiring arteriovenous grafts), or preoperative WBI of <0.6. Preoperative assessments included echocardiography, color Doppler ultrasound examination, and Allen's test to confirm intact palmar arch and normal radial/ulnar arteries. Initial WBI screenings were performed in the clinic before surgery. Patients with a WBI less than 0.6 were excluded for that limb. Values of ≥0.6 qualified for surgery, but these were not recorded in detail and thus could not be correlated with postoperative results. None were <0.6 preoperatively. The WBI was calculated as the ratio of wrist pressure to the highest brachial artery pressure from both arms, measured with validated digital sphygmomanometers. Patients with brachial pressure differences of >20 mm Hg also were excluded, reducing the cohort to 80 patients. The WBI was measured preoperatively, immediately postoperatively, at 2 weeks, and at 3 months. AVF functionality was confirmed intraoperatively by thrill or bruit. Patients were assessed for steal syndrome symptoms (pain, pallor, coldness, numbness, and paresthesia) at each follow-up using a standardized checklist. Temporary steal syndrome was defined as symptoms resolving within 3 months, prolonged steal syndrome as symptoms persisting beyond 3 months, and severe steal syndrome as limb-threatening ischemia requiring surgical intervention.

Patients with AVF failure or immature fistulas were excluded to standardize the cohort. These patients were immediately referred for alternative access, and further WBI measurements were not feasible. Patients who were not able to be followed were also excluded, resulting in 59 patients (Fig). Data were analyzed using SPSS 18 with Shapiro-Wilk, *t* test, and Mann-Whitney *U* tests (*P* < .05). The study was approved by the university's ethics committee.

**Fig:**
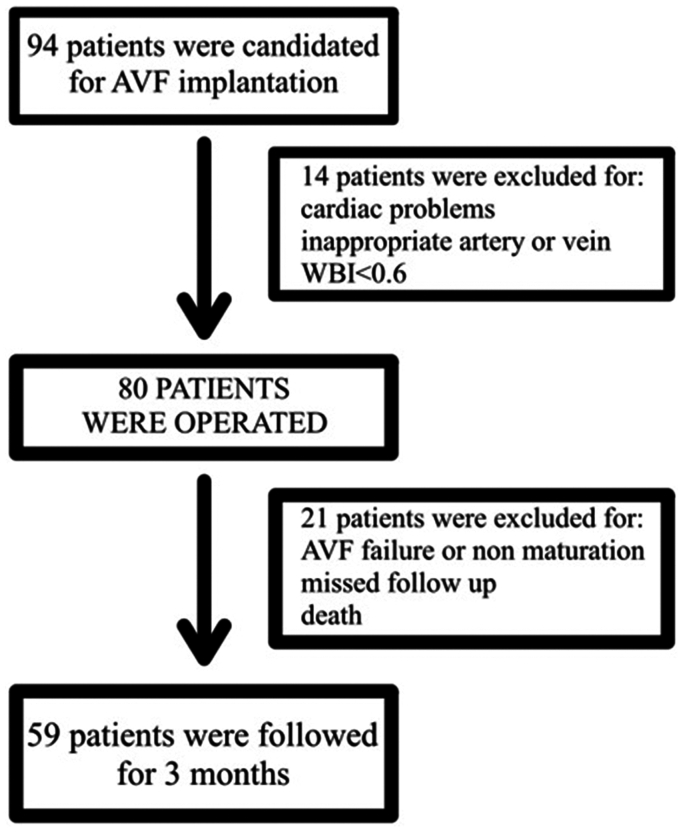
Enrolment flowchart. *AVF*, arteriovenous fistula; *WBI*, wrist-brachial index.

## Results

Of 59 patients (31 men, 28 women; mean age, 56.9 ± 12.6 years), 36 (61%) had diabetes, 33 (55.9%) had hypertension, 16 (27.1%) were smokers, and 33 (55.9%) had other comorbidities (eg, coronary disease, hypothyroidism). Forty-two patients (71.2%) had prior hemodialysis via catheters (mean duration, 35.7 ± 12.6 months). The mean preoperative WBI was 0.95 ± 0.11, decreasing to 0.74 ± 0.19 immediately postoperatively, 0.73 ± 0.17 at 2 weeks, and 0.69 ± 0.19 at 3 months (Table I). WBI changes (ΔWBI = [(Current WBI – Preoperative WBI)/Preoperative WBI] × 100) averaged 22.1%, 23.1%, and 27.4% at early, 2-week, and 3-month intervals, respectively (Table II).

**Table I:** Wrist-brachial index (*WBI*) measures in different time intervals

	The lowest	The most	Average	Standard deviation
WBI before operation	0.71	1.17	0.95	0.11
WBI immediately after the procedure	0.26	1.20	0.74	0.19
WBI 2 weeks after surgery	0.3	1.07	0.73	0.17
WBI 3 months after surgery	0.26	1.33	0.69	0.19

**Table II:** Wrist-brachial index (*WBI*) changes in different time intervals

	Reduction (percent)	No reduction (percent)	Total
WBI changes Immediately	55 (93.22%)	4 (6.77%)	59 (100%)
WBI changes after 2 weeks	53 (89.83%)	6 (10.16%)	59 (100%)
WBI changes after 3 months	56 (94.91%)	3 (5.08%)	59 (100%)

Steal syndrome occurred in 23 patients (39%) at 2 weeks (temporary), 16 (27.1%) at 3 months (prolonged), and 3 (5.1%) with severe ischemia requiring surgical intervention (Table III). Seven patients developed new steal syndrome symptoms at 3 months. Patients were classified into four groups based on the occurrence of steal syndrome. This classification is defined in relation to WBI changes in each group in Table IV. Immediate postoperative WBI changes had significant association with steal syndrome incidence (*P* = .02). Changes in the WBI after 2 weeks and 3 months also correlated with steal syndrome, but these correlations were not meaningful (*P* = .14 and .11, respectively) (Tables IV and V).

**Table III:** The incidence of the different types of steal syndrome

	No.	Percent
Temporary steal syndrome	23	39
Prolonged steal syndrome	16	27.1
Severe steal syndrome	3	5.1

**Table IV:** Patients' classification according to the type of steal syndrome

Group		No.	Percent	Average ΔWBI (percent)		
				After surgery	After 2 weeks	After 3 months
1	No steal	29	49.2	16.66	22.68	26.88
2	Only temporary steal	14	23.7	33.98	21.12	32.94
3	Only prolonged steal	7	11.9	18.74	25.69	27.14
4	Temporary and prolonged steal	9	15.3	63.12	27.71	35.92
Total		59	100			

*WBI,* wrist-brachial index.

**Table V:** The association between wrist-brachial index (*WBI*) changes in different time intervals with the incidence of steal syndrome

ΔWBI	Average incidence of steal syndrome	Standard deviation	*P*-value
Immediately after surgery	26.87%	22.44%	0.02
Two weeks after surgery	25.37%	12.37%	0.14
Three months after surgery	33.97	16.63	0.11

In the severe ischemia subgroup (n = 3), the mean WBI changes were 64.05% (range, 62.23–65.6%) immediately postoperatively, 28.91% (range, 28.19–29.32%) at 2 weeks, and 36.02% (range, 35.23–36.43%) at 3 months. Immediate postoperative WBI changes were significantly associated with severe ischemia (*P* = .03), but not at 2 weeks (*P* = .41) or 3 months (*P* = .38).

By comparing the *P* values in two other periods and considering the increase in WBI changes over time, it can be concluded that the *P* values (.14 to .11) are changing toward the reduction and significance of the association. WBI changes correlated significantly with hypertension (*P* = .01 immediate, *P* = .02 at 3 months) and smoking (*P* = .03 at 3 months), but not with age, sex, dialysis duration, diabetes, or body mass index (Tables VI and VII).

**Table VI:** The association between wrist-brachial index (*WBI*) changes in different time intervals with quantitative variations

	ΔWBI		
	*P* value (correlation coefficient) Immediately after surgery	*P* value (correlation coefficient) Two weeks after surgery	*P* value (correlation coefficient) Three months after surgery
Age	.53 (−0.08)	.85 (−0.02)	.79 (0.03)
Dialysis duration	.50 (0.08)	.51 (−0.08)	.81 (0.03)
BMI	.20 (−0.16)	.84 (−0.02)	.45 (−1.0)

**Table VII:** The association between wrist-brachial index (*WBI*) changes in different time intervals with qualitative variations

Variation	*P* value Early WBI changes	*P* value 2 weeks WBI changes	*P* value 3 months WBI changes
Sex	.11	.10	.07
Smoking	.98	.98	.03
Hypertension	.01	.79	.02
Diabetes	.31	.03	.98

## Discussion

Steal syndrome complicates AVF implantation, with severe cases requiring intervention in <10% of patients.^4^ In this study, 50.8% of patients experienced steal syndrome, but only 5.1% had severe ischemia, consistent with prior reports.^9^ A preoperative WBI of <0.6 contraindicates AVF creation,^13^ yet steal syndrome, including severe cases, can occur in limbs with normal WBI, necessitating postoperative predictors.^17^ Early postoperative changes in the WBI are attributed to flow diversion from the arterial system to the venous system through the fistula and resultant decrease in wrist pressure. Therefore, ischemic symptoms are common in the early postoperative period secondary to a decrease in distal arterial perfusion.^21^^,^^22^ In our study, 23 patients (39%) experienced ischemic symptoms in the early postoperative period.

The significant association with severe ischemia (*P* = .03) underscores the predictive value of early WBI changes. Over time, distal arterial vasodilation reduces peripheral vascular resistance, increasing limb flow despite a lower WBI.^23^^,^^24^ This adaptation explains why late WBI changes (2 weeks: *P* = .41; 3 months: *P* = .38) were less predictive of steal syndrome.

Hypertension and smoking correlated with WBI changes, likely owing to their effects on vascular compliance.^24^ The lack of association with diabetes, age, or body mass index suggests hemodynamic factors primarily drive WBI changes.

### Limitations

The sample size (n = 59) and small severe ischemia subgroup (n = 3) limit statistical power for subgroup analyses. The absence of a validated steal syndrome questionnaire may introduce subjectivity. Future studies with larger cohorts should explore longitudinal WBI trends and additional predictors, such as arterial diameter ratios.^22^

## Conclusions

Although a preoperative WBI of ≥0.6 remains the minimum threshold for AVF creation, significant immediate postoperative WBI reductions are a serious risk factor for steal syndrome and should prompt close follow-up, especially when combined with hypertension or smoking. Immediate postoperative WBI changes reliably predict steal syndrome, including severe cases requiring intervention, after AVF implantation. Patients with significant early WBI reductions, particularly >60%, require close monitoring to detect and manage ischemic complications promptly.

## Funding

None.

## Disclosures

None.
